# The LaserFIB: new application opportunities combining a high-performance FIB-SEM with femtosecond laser processing in an integrated second chamber

**DOI:** 10.1186/s42649-020-00044-5

**Published:** 2020-10-26

**Authors:** Ben Tordoff, Cheryl Hartfield, Andrew J. Holwell, Stephan Hiller, Marcus Kaestner, Stephen Kelly, Jaehan Lee, Sascha Müller, Fabian Perez-Willard, Tobias Volkenandt, Robin White, Thomas Rodgers

**Affiliations:** 1 ZEISS Research Microscopy Solutions, Carl-Zeiss-Straße 22, 73447 Oberkochen, Germany; 2 ZEISS Process Control Solutions, 4385 Hopyard Rd, Pleasanton, CA 94588 USA; 3 ZEISS Research Microscopy Solutions, Cambourne Business Park, Cambourne, Cambridgeshire, UK; 4 ZEISS Process Control Solutions, Carl-Zeiss-Straße 22, 73447 Oberkochen, Germany; 5 Carl Zeiss X-ray Microscopy, 4385 Hopyard Rd, Pleasanton, CA 94588 USA

**Keywords:** LaserFIB, FIB-SEM, PFIB, Crossbeam laser, Femtosecond laser, Dual chamber SEM

## Abstract

The development of the femtosecond laser (fs laser) with its ability to provide extremely rapid athermal ablation of materials has initiated a renaissance in materials science. Sample milling rates for the fs laser are orders of magnitude greater than that of traditional focused ion beam (FIB) sources currently used. In combination with minimal surface post-processing requirements, this technology is proving to be a game changer for materials research. The development of a femtosecond laser attached to a focused ion beam scanning electron microscope (LaserFIB) enables numerous new capabilities, including access to deeply buried structures as well as the production of extremely large trenches, cross sections, pillars and TEM H-bars, all while preserving microstructure and avoiding or reducing FIB polishing. Several high impact applications are now possible due to this technology in the fields of crystallography, electronics, mechanical engineering, battery research and materials sample preparation. This review article summarizes the current opportunities for this new technology focusing on the materials science megatrends of engineering materials, energy materials and electronics.

## Introduction

The development of the femtosecond laser (fs laser) with its ability to provide extremely rapid athermal ablation of materials has initiated a renaissance in materials science. Sample milling rates for the fs laser, summarised in Table 1, are orders of magnitude greater than that of traditional ion sources currently used. In combination with minimal surface post-processing requirements, this technology is proving to be a game changer for materials research.

**Table 1:** Milling rates for the femtosecond laser are 3 orders of magnitude faster than for a Xe + PFIB, enabling new areas of research to be explored. Rates are presented in μm^3^/sec [rates are empirical (this work) unless specified]

Material	Ga FIB	Xe Plasma FIB	fs Laser
Metal (steel)	18	6.7 × 10^2^	2.8 × 10^5^
Tungsten carbide	12^a^	1.3 x 10^2b^	3.3 × 10^5^
Nuclear Graphite	N/A	N/A	3.3 × 10^5^
Silicon	1.0 × 10^2^	6.7 × 10^2^	5.4 × 10^5^

^a^ - (Ali et al. 2005)

^b^ - (Burnett 2016)

In 2012, the first nanosecond laser combined with a focused ion beam scanning electron microscope (FIB-SEM) was introduced by ZEISS, enabling researchers to quickly access deeply buried structures in materials. Although nanosecond lasers are used for laser ablation, their pulse duration permits significant heat transfer to the sample. This creates an unwanted temperature rise at the surface leading to thermal vaporisation, evaporation and plasma formation. This leads to local heating (Hamad 2016) (LaHaye et al. 2013) and subsequent melting of the native microstructure.

Due to the temporal pulse length, this heating effect prevents investigation of surface or near surface microstructure, unless significant material post processing is performed to remove sample damage. The so called ‘heat affected zone’ (HAZ) caused by the nanosecond laser pulse requires a relatively large layer of material (20 μm) to be removed in order to access the pristine microstructure. Whilst this technique has found several key applications, the time required to remove the HAZ prevented widespread adoption in industry for process control or failure analysis and drove interest in femtosecond lasers.

The application of a femtosecond laser in combination with a scanning electron microscope for the preparation of cantilevers for micromechanical testing was first presented by Pfeiffenberger et al. (Pfeifenberger et al. 2017). It was shown that significant increases in throughput were possible making previously impossible experiments easily within the reach of the technique.

With the introduction of a femtosecond laser on a FIB-SEM as described in (Barnett et al. 2020), this HAZ was significantly reduced thanks to the athermal nature of heat transport enabled by the femtosecond laser pulse length. Combined with material removal rates orders of magnitude faster than traditional methods such as gallium FIB or plasma FIB (PFIB) milling (Table 1), this new LaserFIB enables numerous new capabilities, including access to deeply buried structures as well as production of extremely large trenches, cross sections, pillars, and TEM H-bars, all while preserving microstructure and avoiding or reducing FIB polishing.

A number of high impact applications are now possible due to this technology in the fields of crystallography, electronics, mechanical engineering, battery research and materials sample preparation.

### The LaserFIB concept

**Fig. 1:**
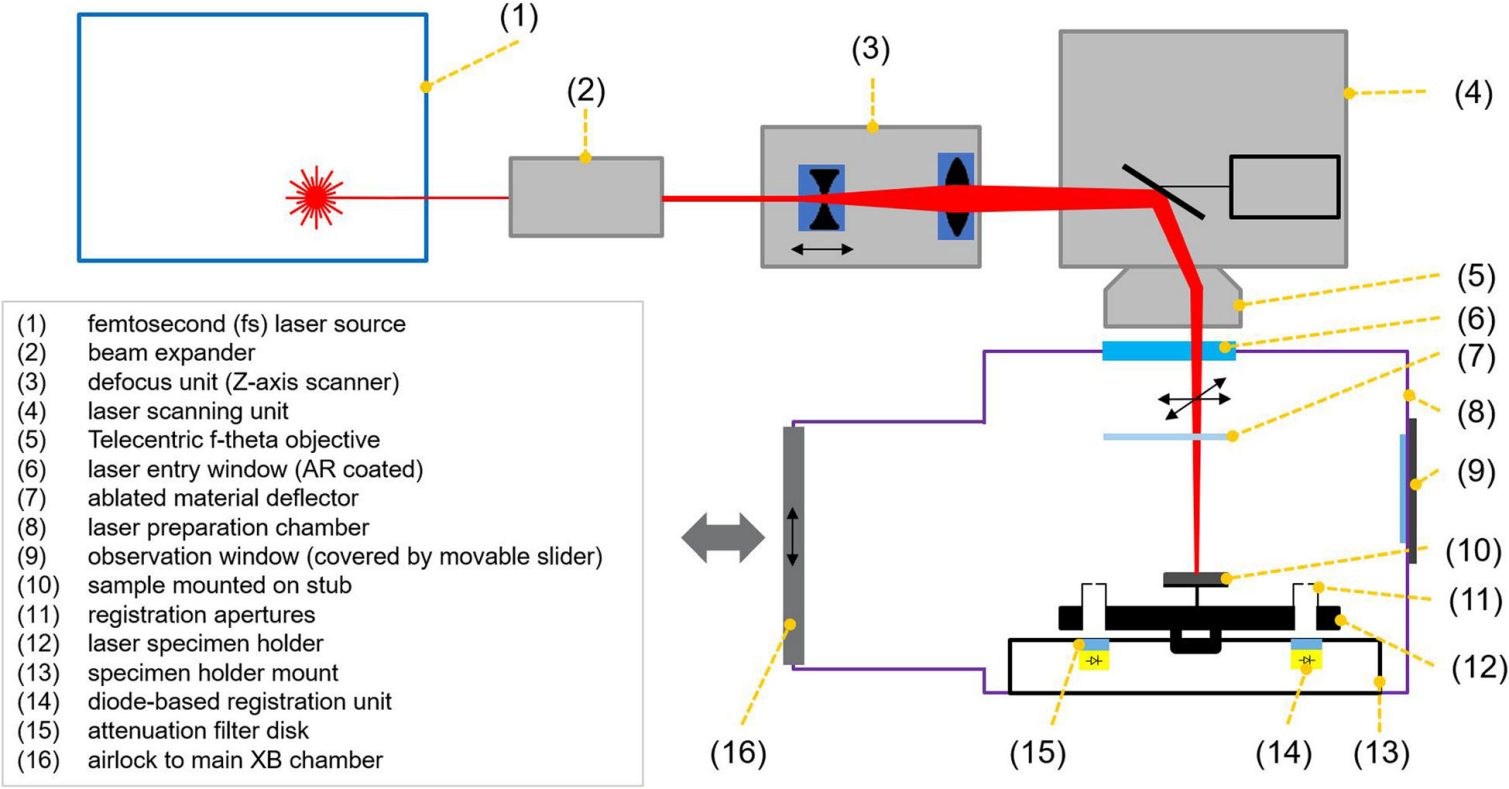
Schematic layout of the fs laser chamber with principal components outlined

The ZEISS Crossbeam laser system described in Fig. 1 and Table 2 employs a dual chamber system with a high-resolution FIB-SEM (ZEISS Crossbeam 350 or ZEISS Crossbeam 550) coupled to a connected second laser processing chamber. The dual chamber approach has several advantages:

1. Material removal takes place outside of the main FIB-SEM chamber. This has the advantage of containing the ablated debris away from the high-resolution imaging chamber, which eliminates the risk of damage to precision components and prevents contamination that can reduce the instrument’s operational resolution.
2. The electron imaging beam, gallium FIB beam and fs laser beam are not coincident on a single point. A separate chamber dedicated to laser processing allows for large areas of the sample to be accessed. The laser scanning module enables a total area of 40 mm × 40 mm to be interrogated by the laser. This ensures large arrays of repeating structures can be fabricated for experiments requiring larger statistical datasets for better representivity.
3. It is possible to configure up to 10 detectors onto the main FIB-SEM chamber whilst still having the fs laser connected to the adjacent chamber. This enables unique application capabilities such as femtosecond laser preparation of a large surface followed by secondary ion mass spectrometry (SIMS) analysis of the surface without exposing the surface to air. This setup also benefits from a Ga + FIB source, resulting in several SIMS advantages over PFIB sources, with the principal advantage being the greater spatial resolution of a Ga source.
4. An advanced laser scanning module can be implemented. The additional space afforded by the second chamber enables the addition of a telecentric lens system to ensure that the laser light is incident perpendicular to the sample surface across the entire 40 mm × 40 mm scan field. In a coincident geometry setup, such a design would not be feasible due to the limited space available in the chamber. The result is that the ablation direction is independent of the laser beam position on the scan field and full control of the laser ablation process is ensured.
5. Femtosecond laser processing of materials can be performed simultaneously to work being done in the main chamber, thus increasing system productivity.

**Table 2:** ZEISS Crossbeam laser femtosecond laser operating parameters

ZEISS crossbeam laser specifications	
Supplier	TRUMPF GmbH + Co. KG
Type	Diode pumped solid state fiber laser
Wavelength (λ)	515 nm (green)
Optics	telecentric, f = 100 mm
Pulse Duration	< 350 fs
Pulse repetition rate	1 kHz - 1 MHz
Max. average power	10 W @ 1 MHz
Max. pulse energy	10 μJ @ 1 MHz
Peak laser power:	max. 20 MW
Beam quality	TEM00 (M^2^ < 1,3)
Focus tracking length	6 mm (+/−3 mm)
Focal diameter (spot)	14 μm
Rayleigh length	150 μm
Scan field size	40 × 40 mm^2^

With these significant advantages, a number of challenges remain for the technique, such as:

1. By removing volumes of material up to cubic millimetres, the proper handling of such material is critical. The ablated material forms a plume normal to the sample surface and energetic ablation products coat the laser entry window (Fig. 1, component 6) unless the material can be directed away from the laser entry window. The coating of this window reduces the transmission of the laser light and eventually renders the instrument unusable without stopping the experiment and cleaning the optical component affected. This can be completely mitigated through the use of an ablated material deflector (Fig. 1, component 7) device. This device is intentionally not described here in detail due to its commercial sensitivity.
2. The laser ablation chamber requires cleaning periodically. Once the ablated material has been deflected, it generally settles on the chamber surfaces. This requires periodic (weekly or monthly depending on use) cleaning of the chamber walls with a solvent depending on the material being ablated. A sacrificial chamber sleeve can be implemented to make this process fast and simple.
3. The laser ablation causes Laser Induced Periodic Surface Structures (LIPSS) to form on the surface of the sample. These structures are dependent upon the polarization of the laser but can be removed with a gallium ion beam polish. A mechanical polarization rotator can also be used to mitigate this which averages the directionality of the laser polarization to zero which in turn minimizes the LIPSS effect.
4. Moving the sample between the main chamber and the laser ablation chamber has a repeatability error of approximately 2 μm meaning repetitive workflows must work within this limit. This is actually extremely accurate since the laser spot size is of the order of 15 μm.

### Applications for electron backscatter diffraction

Electron backscatter diffraction (EBSD) has become a ubiquitous technique in scanning electron microscopy. Local diffraction patterns are generated and used to determine grain structure, orientation, phase and strain. With a surface sensitivity of 10–50 nm, the technique accordingly requires a smooth surface to produce high-quality diffraction patterns. The requirement to be free from contamination, damage or oxidation, is enabled by milling via focused ion beam (FIB) or laser within a vacuum.

Sample preparation of buried structures for EBSD, such as electronics assemblies or inclusions in metals, is time consuming through conventional means such as grinding, polishing or ion milling. There is also the risk that the sample can pick up contamination or corrosion during transfer operations. It is therefore advantageous to enable rapid material removal under vacuum within the second chamber, achieving minimal collateral damage and acceptable surface roughness for EBSD (less than 10 nm).

**Fig. 2:**
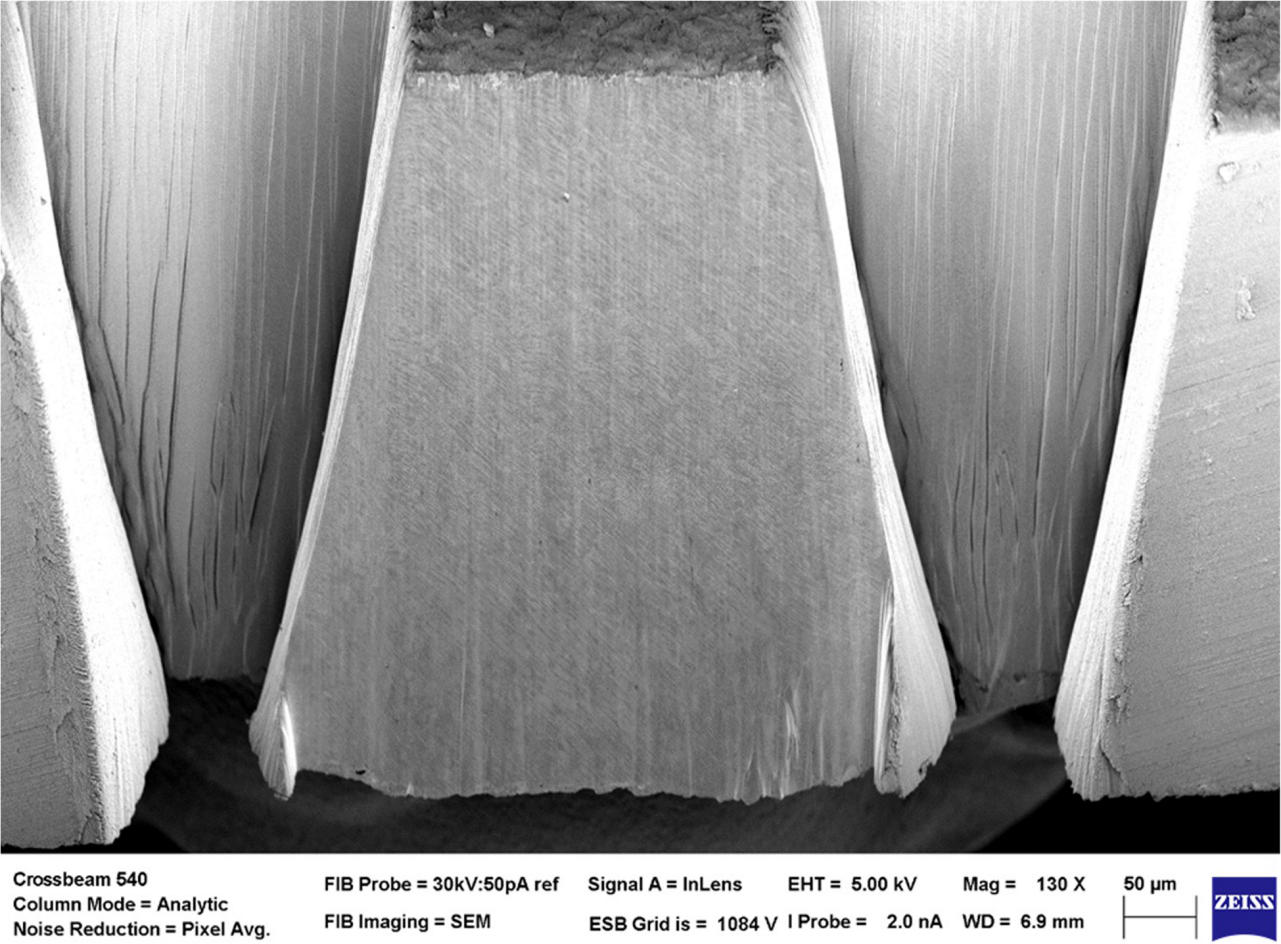
Direct observation of a large 3D structure produced from direct fs laser ablation. The surface of the sample shows some minor repeating structures called LIPSS. Such 3D structures can then be used as a starting point for high resolution 3D tomography or EBSD using standard FIB-SEM workflows

Femtosecond laser ablation is known to produce LIPSS through mechanisms debated extensively in the literature (Gurevich 2016). LIPSS can hinder microstructural visualisation and image analysis, as well as affect resolution of EBSD patterns. However, moderate LIPSS are not obstructive to adequate EBSD pattern resolution as the EBSD sampling depth (up to 50 nm) is greater than the surface variation, and novel method development has been carried out to limit formation of LIPSS to within acceptable boundaries. Although often visible, LIPSS do not necessarily inhibit EBSD pattern resolution. Figure 2 show the quality of a laser processed surface without any post polishing. The underlying microstructure can clearly be seen.

State-of-the-art EBSD cameras are also tolerant to noisier Kikuchi patterns, and associated software packages can sufficiently process data such that the fs-laser ablated surfaces are smooth enough for full EBSD resolution without the need for a time-consuming FIB polishing step. This approach is described in detail elsewhere (Schubert 2020).

Prior to the advent of the fs laser, deeply buried strata or inclusion fields would require time consuming FIB milling or a multi-stage process of cutting, polishing and FIB milling to enable access and perform EBSD analyses. It is therefore advantageous to directly access deeply buried features for 2D or 3D EBSD using a rapid ablation with acceptable surface quality. Hence, the possibility of rapid direct EBSD preparation via femtosecond laser ablation without the need for further surface improvement via FIB milling delivers a consequent dramatic reduction in preparation time.

A rough laser machining step at high power is used for initial mass ablation, followed by one or two fine polishing steps using the femtosecond laser at optimised parameters of laser power, frequency, traverse speed and line spacing. A laser milling regime has been developed whereby no further FIB or other polishing is required to further mill the surface for EBSD.

Metal microstructure is visible from the raw lasered surface, including sulfide and oxide inclusions in steel. Laser milling parameters are equally and highly effective on several common engineering alloys including steel, copper, alloy 600 nickel superalloy and rolled aluminium.

**Fig. 3:**
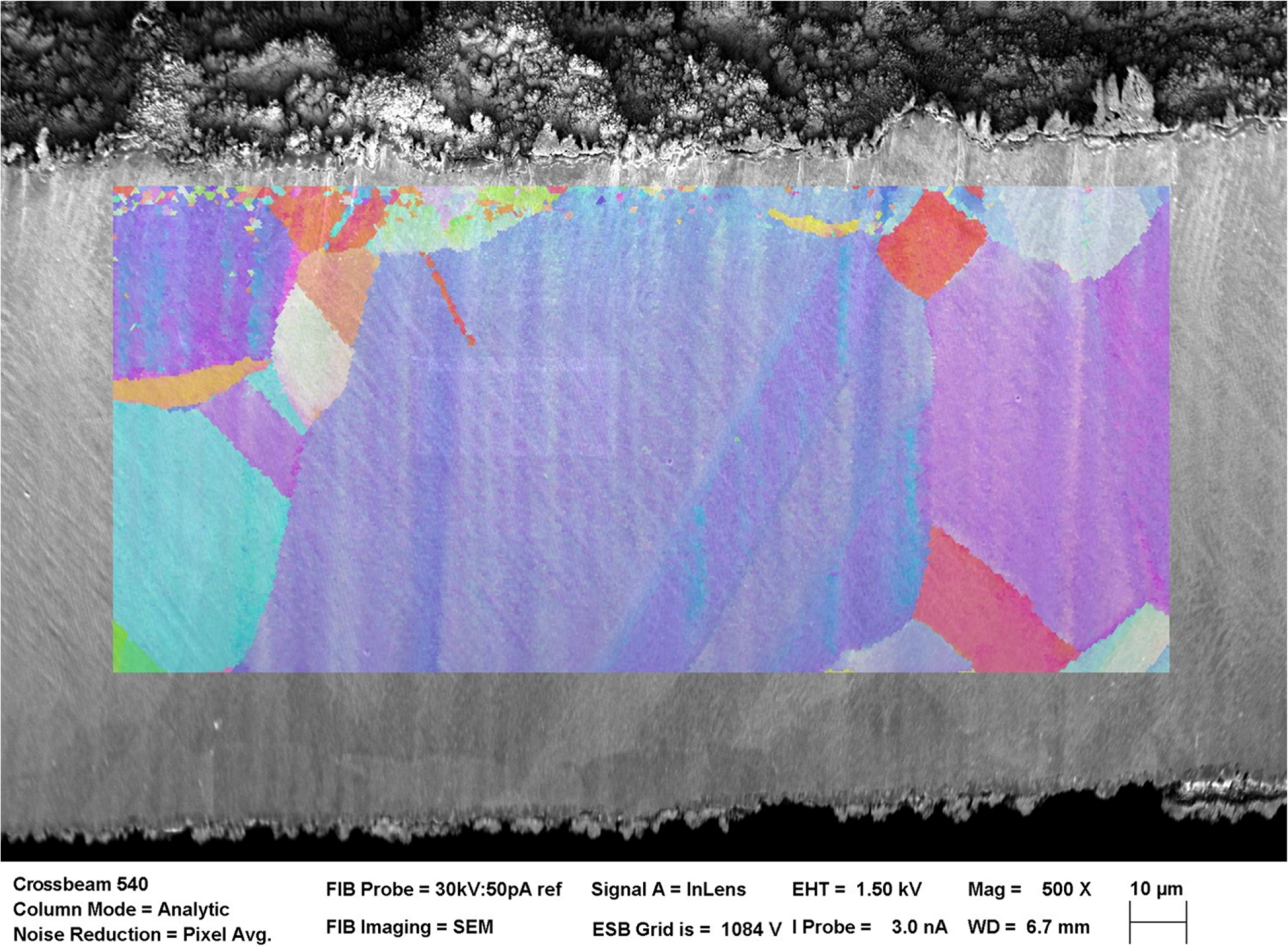
EBSD grain map overlaid on laser milled surface of a copper sample. The laser milled surface is sufficient to produce adequate indexing to enable EBSD mapping

By reducing processing to two passes of the laser at optimised parameters, a total of only 140 s of surface preparation time can be achieved for a cross section in excess of (500 μm)^2^. Although LIPSS and polishing artefacts are found to affect the EBSD signal and create some unresolved pixels, further post processing can be used to resolve missing pixels (Schubert 2020). Furthermore, copper, steel and nickel superalloy samples generated acceptable EBSD patterns without appreciable image post processing as shown in Fig. 3.

Therefore, high throughput alloy and inclusion research or quality assurance by EBSD of metallic or other surfaces is enabled by extremely rapid sample preparation using the femtosecond laser, as well as serial slicing over large volumes (Echlin 2020) for location and characterization of buried features such as non-metallic inclusions and other buried structures.

### Applications in the study of hard materials and ceramics

**Fig. 4:**
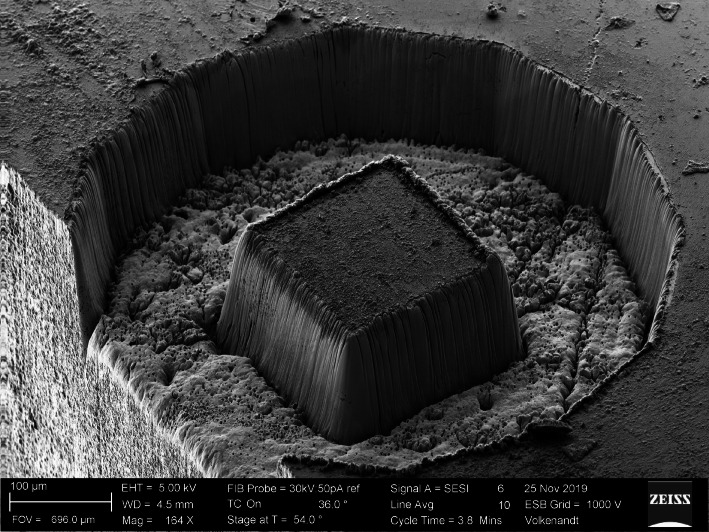
Scanning electron micrograph of a WC island milled into a bulk sample surface. The island is 180 μm on wide and 120 μm high and was milled in 85 s using the ZEISS Crossbeam Laser

**Fig. 5:**
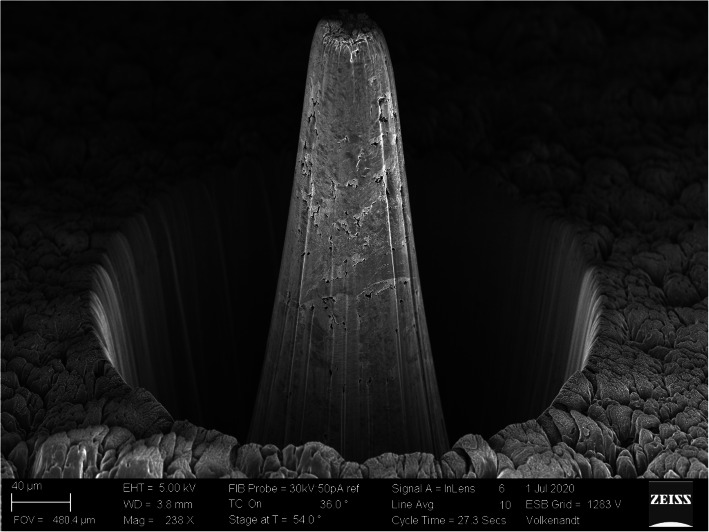
Micropillar of nuclear grade graphite prepared using the fs laser ablation attachment of the ZEISS Crossbeam. The pillar protrudes 250 μm above the sample surface of a ~ 1 mm^2^ bulk sample. The laser milling time was 750 s

Ion beam milling of hard and/or dense materials poses challenges. For modest material removal volumes of ~[10 μm]^3^ the problem is easily solvable with longer instrument milling times. However, for applications where large amounts of material need to be removed (more than ~[100 μm]^3^), the problem becomes intractable. For example, recent reports (Burnett 2016) of Xe^+^ plasma-FIB milling of a WC-Co hard metal reported a milling rate of 134 μm^3^/sec. To remove a volume of 1 mm^3^ at this rate would take over 86 days, while fs laser milling can remove similar volumes in a few hours. This can be seen visually in Fig. 4 where the isolated 180 μm wide WC island was milled into the sample surface in 85 s. Similarly, nuclear-grade graphite is difficult to mill using either Ga^+^ or Xe^+^ based ion beam techniques because of both its extreme hardness and the atomic makeup. This material can be milled easily with fs laser ablation, as demonstrated in Fig. 5.

The x-ray nanotomography pillar shown in Fig. 5 was milled by removing all surrounding material on a 1 mm × 1 mm bulk sample to a depth of 250 μm with 750 s of laser milling time. This equates to a removal rate of over 1 mm^3^ per hour, more than 2000 times faster than possible with a plasma FIB.

Additionally, insulating materials such as ceramics do not suffer from poor milling performance with fs laser ablation as they do with Ga + or Xe + based ion beam techniques. Because the laser uses photons to mill the material instead of charged particles, sample charging does not affect the interaction of the laser beam with the material. In this manner, insulating materials such as glasses or oxides as well as materials with poor electrical connections to their substrates can be effectively milled using fs laser ablation to reveal deeply buried structures. Furthermore, the femtosecond laser can effectively ablate wide bandgap materials since multiphoton ionization can lead to the effective absorption of laser energy. This property promises to bring wide applicability to ceramics research.

### Correlative microscopy for guided sample preparation

Many materials analysis challenges require connecting data across different length scales and analytical modalities to form a comprehensive understanding of the mechanisms at play or to solve a processing challenge (A. Poozhikunnath et al. 2019) (Moniri et al. 2020). As an example, 3D pore networks in battery electrodes may vary across distances of centimeters to millimeters, while the details of the networks that affect charge and fluid transport depend on the local microstructure down to the nanometer scale (Shearing et al. 2012) (Daemi et al. 2018). Furthermore, charge transfer efficiency and ageing mechanisms may depend on local chemistry and crystallography.

The new LaserFIB provides a targeted, connected route to revealing deeply buried structures inside materials and devices. When coupled with cutting edge non-destructive 3D imaging techniques like x-ray microscopy (e.g., with ZEISS Xradia Versa), this capability enables new multiscale, multimodal, correlative analytical workflows to tackle advanced materials analysis problems. Several previously cumbersome or impractical workflows now become possible using this new capability.

A workflow where the LaserFIB is applied to a 3D microscale x-ray microscopy sample to prepare it in a matter of minutes for subsequent 3D x-ray nanotomography analysis could benefit multiscale analysis challenges in highly porous media like polymer electrolyte fuel cells or filtration media. Corrosion or fatigue research could benefit from a workflow leveraging unique x-ray microscopy contrast modalities such as Laboratory Diffraction Contrast Tomography followed by a LaserFIB step to expose a deeply buried failure site for analysis with high resolution EBSD. For atomic level imaging modalities such as transmission electron microscopy or atom probe tomography (APT), buried features of interest can be targeted precisely based on micro- or nano-scale x-ray microscopy data, and the LaserFIB provides rapid access to these buried structures for precise, targeted sample preparation with the Ga FIB. Moreover, atom probe tomography samples can be prepared without using the lift-out technique, as the fs laser can create a pillar and then increase its prominence from the surface by milling the entire surface area away to reveal a protruding APT tip. Sample preparation is faster than the lift-out method, and it removes a problematic interface that can cause failures during atom probe analysis. There are also opportunities for a correlative workflow from 3D x-ray nanotomography imaging on the order of 16 nm voxel size combined with chemical information from APT.

### Opportunities in electronics research and production

Electronics are powered by sophisticated semiconductor devices, and increasingly, the semiconductor package is an important driver of device and system performance. Semiconductor devices, inclusive of packaging, use a vast array of periodic table elements in the effort to optimize for higher functionality, lower power consumption, higher speed and lower latency (Boyd et al. 2007). At the same time, features are shrinking while also being stacked, resulting in high interconnect densities.

**Fig. 6:**
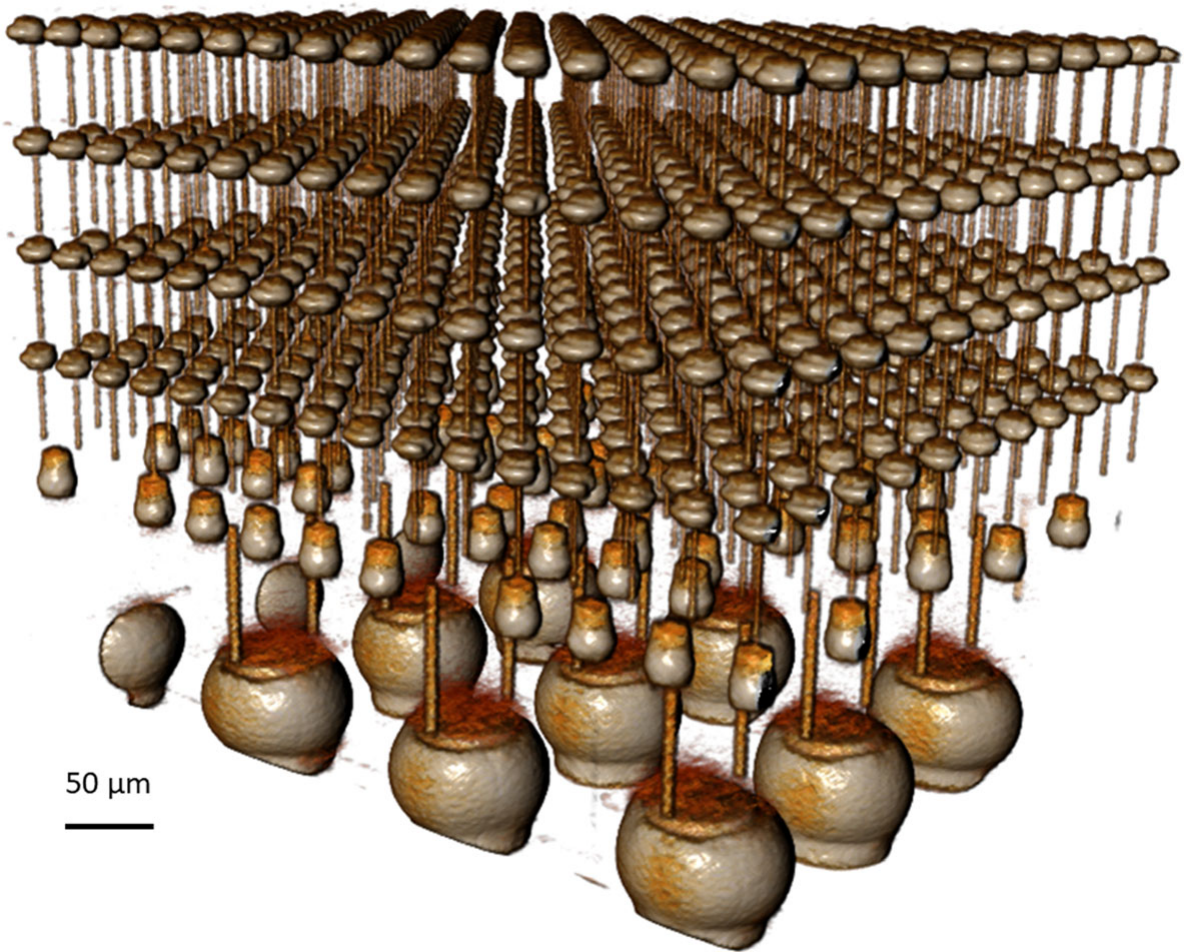
3D X-ray image of a < 1 mm field-of-view of package interconnect structures within an AMD Vega64 2.5D package. Acquired with ZEISS Xradia 620 Versa using 0.7 μm voxel size

Figure 6 is a 3D X-ray microscope image of the high-bandwidth memory (HBM) portion of an AMD Vega64 2.5D package, showing state-of-the-art package interconnect complexity. It is increasingly challenging to efficiently and accurately characterize assembled structures and their defects, with minimal artefacts and high throughput. This challenge is compounded by the requirement for successful sample preparation, across the vast array of materials used in IC devices and packages, to aid fast development and time to market for new technologies.

**Fig. 7:**
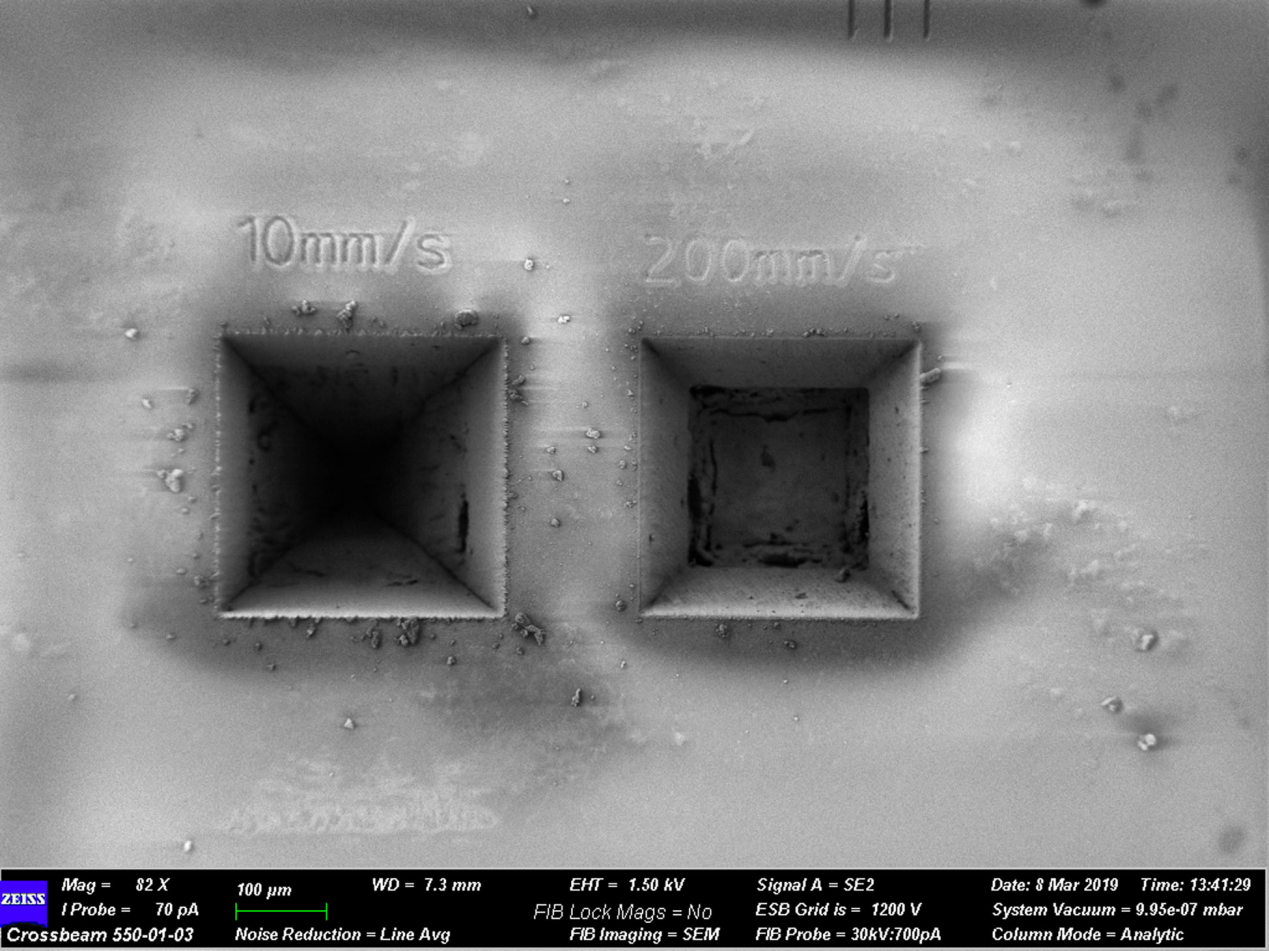
Results of fs-laser ablation of glass processed in 651 and 96 s, respectively

The complexity of electronics makes the integrated fs-laser a particularly effective choice for a site-specific FIB cross-section workflow for rapid analysis of buried structures, since a wide range of materials can be efficiently processed over millimetres of volume with minimal HAZ. This includes polymers, glass, thermal interface material, silicon carbide, mould compound and underfill, multilayer laminated substrates, metals, ultra-low K dielectric materials, and more. Figure 7 shows the effectiveness of the system on glass. Several hundred cubic microns of material on the order of ~ 300 μm × 300 μm × 900 μm were removed in 96 and 651 s, respectively, using ablation conditions for high quality edges.

**Fig. 8:**
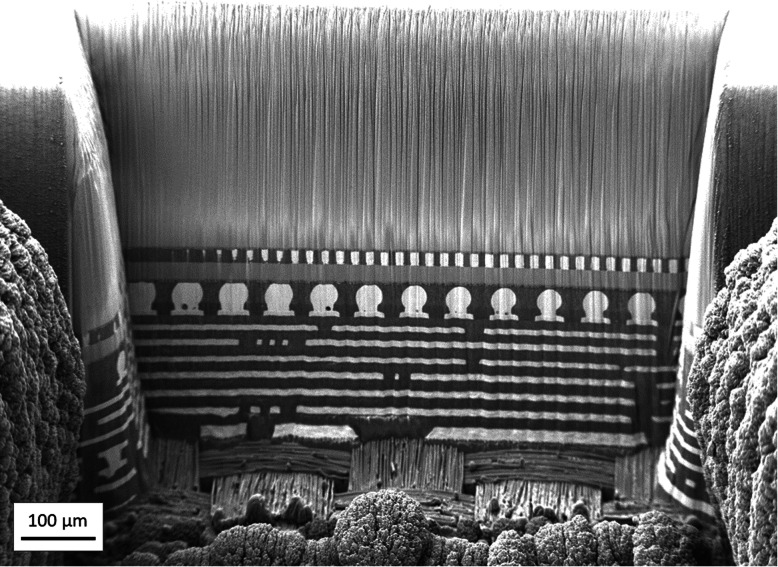
SEM cross-sectional image of a 14 nm silicon node 3D flip chip package immediately after laser polishing, without any ion beam clean-up

The LaserFIB delivers rapid, artifact-free processing of Cu/low K advanced node Si die, as well as package polymeric materials and substrates. Figure 8 shows that with optimized fs-laser polishing under vacuum, structures are immediately visible, aiding visualization to target locations for higher-quality FIB polishing and SEM imaging. The laser affected zone typically penetrates less than half a micron beyond the ablated surface for all materials throughout the depth of heterogeneous advanced Si node 3D packages in this class. The integration of a fs-laser with FIB-SEM enables rapid set-up of the optimized recipes that balances speed with high surface quality and minimal redeposition in a series of “test and view” learning cycles. Paired with high-resolution 3D X-ray data for localization, one can cross section a specific targeted 5 μm void in a 3D package (Kaestner et al. 2019).

**Fig. 9:**
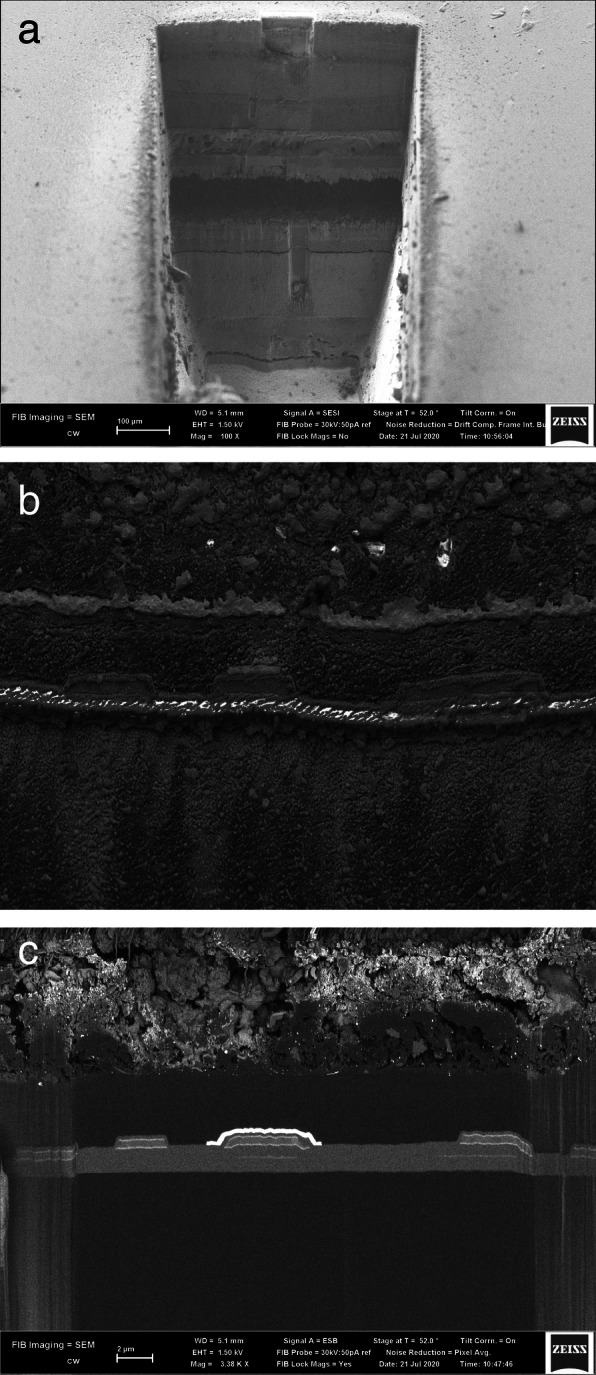
**a** Delamination due to mechanical stress of the active layer of an OLED display - the organic layer and thin-film transistor are found 300 μm below the surface of the device, with 20 s of laser milling. **b** Electrodes of the thin-film transistor layer of a commercial OLED device, after 40 s of very low power fine polishing with the fs-laser. **c** Electrodes of the thin-film transistor layer of a commercial OLED display, after FIB polishing for 10 min

In the consumer market for OLED displays, it is often instructive to access the TFT and active layers of the device at or near a site of failure. Common failure modes include dye fading, encapsulation leaks, and delamination of the organic layers from the electronic backplane. The integrated fs-laser FIB-SEM, through its optimized workflow, prevents the exposure of highly reactive active layers to water content in air, which can confuse the analysis of real failure modes. The laser ablation requires only seconds, and integration achieves fast setup for FIB polishing and SEM imaging. Samples may be processed with resulting images within 1 to 2 h, which is fast enough to avoid vacuum-induced delamination artefacts of organic layers. Figure 9 shows examples of a smartwatch OLED display showing active layer delamination failure due to mechanical stress (a), after fine laser polishing of only a few seconds which allows a thin film transistor to be directly imaged prior to FIB (b) and after FIB polishing (c) (Hare 2017).

## Conclusion

A femtosecond laser-integrated FIB-SEM, or LaserFIB, has been introduced. The LaserFIB represents a step change in the ability to process a wide range of materials extremely rapidly. The speed of removal is orders of magnitude faster than focused ion beams can achieve, enabling completely new workflows and experiments to be envisioned. In parallel, these fast material removal rates are not strong functions of material hardness, making the technique applicable to cutting edge materials systems such as ceramics, thermal barrier coatings and nuclear materials. These properties, combined with an air-free transfer to a FIB-SEM, open new areas of research previously deemed impossible. Proven application across a wide range of materials science include rapid EBSD sample preparation for advanced alloy development, sample preparation for X-ray microscopy, accessing deeply buried structures for correlative workflows and the analysis of air-sensitive pristine battery surfaces.

Perhaps the most immediate opportunities lie in the electronics industry. In the future, continued improvements in 3D X-ray microscopy and LaserFIB technologies, enhanced by advances in machine learning algorithms for better image quality and higher throughput, will enable correlated 3D X-ray imaging and LaserFIB analysis to become an efficient and commonplace capability for a wide range of applications including defect inspection and analysis, construction analysis or reverse engineering, atom probe sample preparation and even process control.

## Data Availability

The data presented in this article can be made available upon request to the corresponding author of this article.

## Abbreviations

AMD: Advanced micro devices (Company name)
APT: Atom probe tomography
EBSD: Electron backscatter diffraction
FIB-SEM: Focused ion beam scanning electron microscope
Fs laser: Femtosecond laser
HAZ: Heat affected zone
HBM: High bandwidth memory
LaserFIB: ZEISS Crossbeam 350 or 550 Laser focused ion beam scanning electron microscope
LIPSS: Laser induced periodic surface structure
Low k: Low dielectric constant
OLED: Organic light emitting diode
SIMS: Secondary ion mass spectroscopy
TEM: Transmission electron microscope
TFT: Thin film transistor
